# Digital nonlinearity compensation in high-capacity optical communication systems considering signal spectral broadening effect

**DOI:** 10.1038/s41598-017-12614-x

**Published:** 2017-10-11

**Authors:** Tianhua Xu, Boris Karanov, Nikita A. Shevchenko, Domaniç Lavery, Gabriele Liga, Robert I. Killey, Polina Bayvel

**Affiliations:** 1 School of Engineering, University of Warwick, Coventry, CV4 7AL United Kingdom; 2 Optical Networks Group, Department of Electronic & Electrical Engineering, University College London, London, WC1E 7JE United Kingdom

## Abstract

Nyquist-spaced transmission and digital signal processing have proved effective in maximising the spectral efficiency and reach of optical communication systems. In these systems, Kerr nonlinearity determines the performance limits, and leads to spectral broadening of the signals propagating in the fibre. Although digital nonlinearity compensation was validated to be promising for mitigating Kerr nonlinearities, the impact of spectral broadening on nonlinearity compensation has never been quantified. In this paper, the performance of multi-channel digital back-propagation (MC-DBP) for compensating fibre nonlinearities in Nyquist-spaced optical communication systems is investigated, when the effect of signal spectral broadening is considered. It is found that accounting for the spectral broadening effect is crucial for achieving the best performance of DBP in both single-channel and multi-channel communication systems, independent of modulation formats used. For multi-channel systems, the degradation of DBP performance due to neglecting the spectral broadening effect in the compensation is more significant for outer channels. Our work also quantified the minimum bandwidths of optical receivers and signal processing devices to ensure the optimal compensation of deterministic nonlinear distortions.

## Introduction

Optical fibre networks form the major part of the current communication infrastructure and carry most of the digital data generated. The increasing demand for higher information rates has led to the development and application of successive generations of ever higher-order modulation formats, denser wavelength division multiplexing (WDM) and more advanced digital signal processing (DSP) in optical communication networks^1,2^. Nyquist-spaced transmission and DSP-based impairment compensation have been developed for effectively enhancing the spectral efficiency and reach of optical communication systems^3,4^. Since linear impairments such as chromatic dispersion (CD), polarisation mode dispersion (PMD), and laser phase noise can be well compensated using DSP, the Kerr fibre nonlinearity and its interaction with amplified spontaneous emission (ASE) noise from optical amplifiers determines the current limitations in modern optical communication systems^5–7^. With the increase of optical power, fibre Kerr nonlinearities lead to a significant performance degradation in communication systems due to both intra-channel and inter-channel nonlinear distortions. The Kerr effect also leads to broadening of the spectrum of the signal propagating in fibre^8–11^, arising from the intra- and inter-channel signal nonlinear interactions^10–14^. Apart from causing performance degradation, the presence of spectral broadening causes the uncertainty in the design of the appropriate matched receiver filters for optimum detection and the calculation of achievable spectral efficiency in optical communications^15–18^. The spectral broadening effects have been investigated in optical communication systems without using any nonlinearity compensation. The power spectral density of XPM-induced spectral broadening has been analysed in refs^19,20^, and the impact of spectral broadening on the transmission performance of WDM systems has been evaluated in refs^12,21^. An in-line compensation of the spectral broadening in standard single mode fibre (SSMF) using dispersion compensating fibre was also demonstrated in the dispersion-managed systems^22^. However, the spectral broadening effect in nonlinearity-compensated transmission systems and its impact on the nonlinearity compensation have never been reported.

Multi-channel digital back-propagation (MC-DBP) has been validated as a promising approach for compensating both intra-channel and inter-channel fibre Kerr nonlinearities in optical communication systems^23–26^. The performance of MC-DBP has been investigated in both single-channel and multi-channel optical transmission systems, where a digital filter is applied before MC-DBP module to select the back-propagated bandwidths and remove the unwanted ASE noise^26–31^. However, the spectral broadening effect is typically not considered in the nonlinearity compensation, and the maximum back-propagated bandwidths for full-field DBP (FF-DBP) are usually set to be equal to the transmitted signal bandwidth^27–31^. Related to the back-propagated bandwidth issue, the oversampling rate, at which DBP is operated, has also been investigated in single-channel quadrature phase shift keying (QPSK) and 5-channel 16-ary quadrature amplitude modulation (16-QAM) transmission systems^24,31,32^, where the required minimum oversampling rates have been studied for a full compensation of the deterministic nonlinear distortions. Nevertheless, even when the system components (optical receiver, add-drop multiplexers, or DSP) guarantee a sufficient oversampling rate and bandwidth window (according to transmitted signals), the performance of FF-DBP for nonlinearity compensation may still be sub-optimal due to the truncation of spectral components in the presence of this spectral broadening. Interestingly, the impact of signal spectral broadening on the performance of MC-DBP has never been quantified and must be considered to explore the effectiveness of nonlinearity compensation, channel tailored receiver design, and the estimation of achievable spectral efficiency.

In this paper, the performance of MC-DBP is investigated for compensating fibre nonlinearities in Nyquist-spaced optical communication systems, where the effect of signal spectral broadening is taken into account. Numerical simulations have been carried out in 32-Gbaud single-channel/multi-channel Nyquist-spaced WDM standard single-mode fibre (SSMF) transmission system. A variety of modulation formats including dual-polarisation QPSK (DP-QPSK), DP-16QAM, and DP-64QAM have been considered to study the impact of modulation format on spectral broadening. It is found that signal spectral broadening must be taken into account to achieve optimal nonlinearity mitigation, for which the full detection of broadened signal spectrum is required. This applies to both single-channel and multi-channel optical communication systems, and is independent of signal modulation formats. For multi-channel systems, the degradation of MC-DBP performance, when the spectral broadening effect is not considered in the nonlinearity compensation, is more significant for outer channels. For single-channel system, the penalty in terms of the best achievable signal-to-noise ratio (SNR) is 2.8 dB for 800 km transmission, and 1.8 dB for 2000 km transmission. For the outer channels in the 5-channel system, the SNR degradation is 1.9 dB for 800 km transmission and 1.0 dB for 2000 km transmission. In addition, the signal spectral broadening effect was evaluated numerically for transmitted signal bandwidths varying from 32 GHz to 800 GHz. It is shown that optical receiver and signal processing devices require additional bandwidth (32 GHz–48 GHz in our scheme) beyond the transmitted signal bandwidth to guarantee complete compensation of deterministic nonlinear distortions in Nyquist-spaced optical transmission.

**Figure 1:**
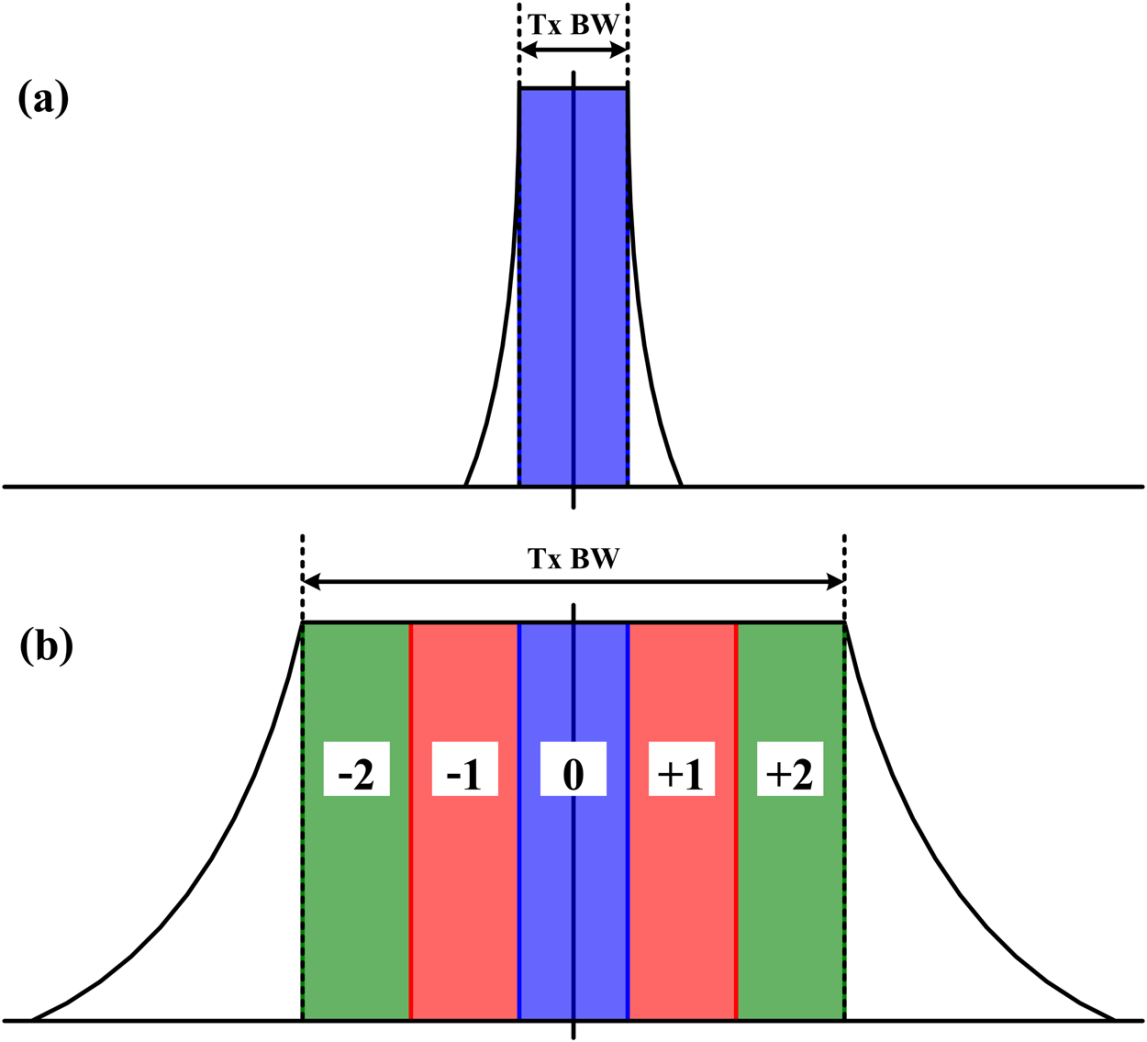
Schematic of signal spectral broadening effect in Nyquist-spaced optical communication systems. (**a**) Single-channel system, (**b**) 5-channel system. Tx BW: transmitted bandwidth.

Self-phase modulation (SPM), cross-phase modulation (XPM) and four-wave mixing (FWM) are the Kerr nonlinear effects in which the phase of the signal and the newly generated frequency components are modulated by the optical power in the fibre due to the intensity dependence of the fibre refractive index^10,33^. These phenomena cause spectral broadening of densely multiplexed optical signals during fibre transmission and induce nonlinear interference between WDM channels^10–12^. Meanwhile, fibre dispersion has a strong impact on the degree of nonlinearity-induced spectral broadening^14^. The effects and changes in nonlinearity-broadened optical spectra in WDM multi-span communication systems depend on the joint interaction between fibre nonlinearity and dispersion^14,34^. The spectral broadening effect has been studied in dispersion-managed optical communication systems, in which significant degradations on the transmission distance and bit-error-rate arose from this effect^9,12^. In this work the effect of spectral broadening was investigated and analysed in dispersion-unmanaged optical communication systems, when the MC-DBP is applied. The schematic of signal spectral broadening effect in single-channel and 5-channel Nyquist-spaced optical communication systems is illustrated in Fig. 1. When the spectral broadening effect is neglected, the optimum back-propagated bandwidth is the same as the transmitted signal bandwidth, since this enables the complete compensation of SPM, XPM and FWM, as well as the removal of out-of-band ASE noise. However, in the presence of spectral broadening, the use of a bandwidth in MC-DBP equal to that of the transmitted signal will lead to a penalty on the performance of nonlinearity compensation due to the truncation of the broadened spectrum. According to Fig. 1(b), the degradation in the nonlinearity compensation in the 5-channel Nyquist-spaced communication system will be more significant for the outer channels due to a more serious truncation of the broadened spectrum.

**Figure 2:**
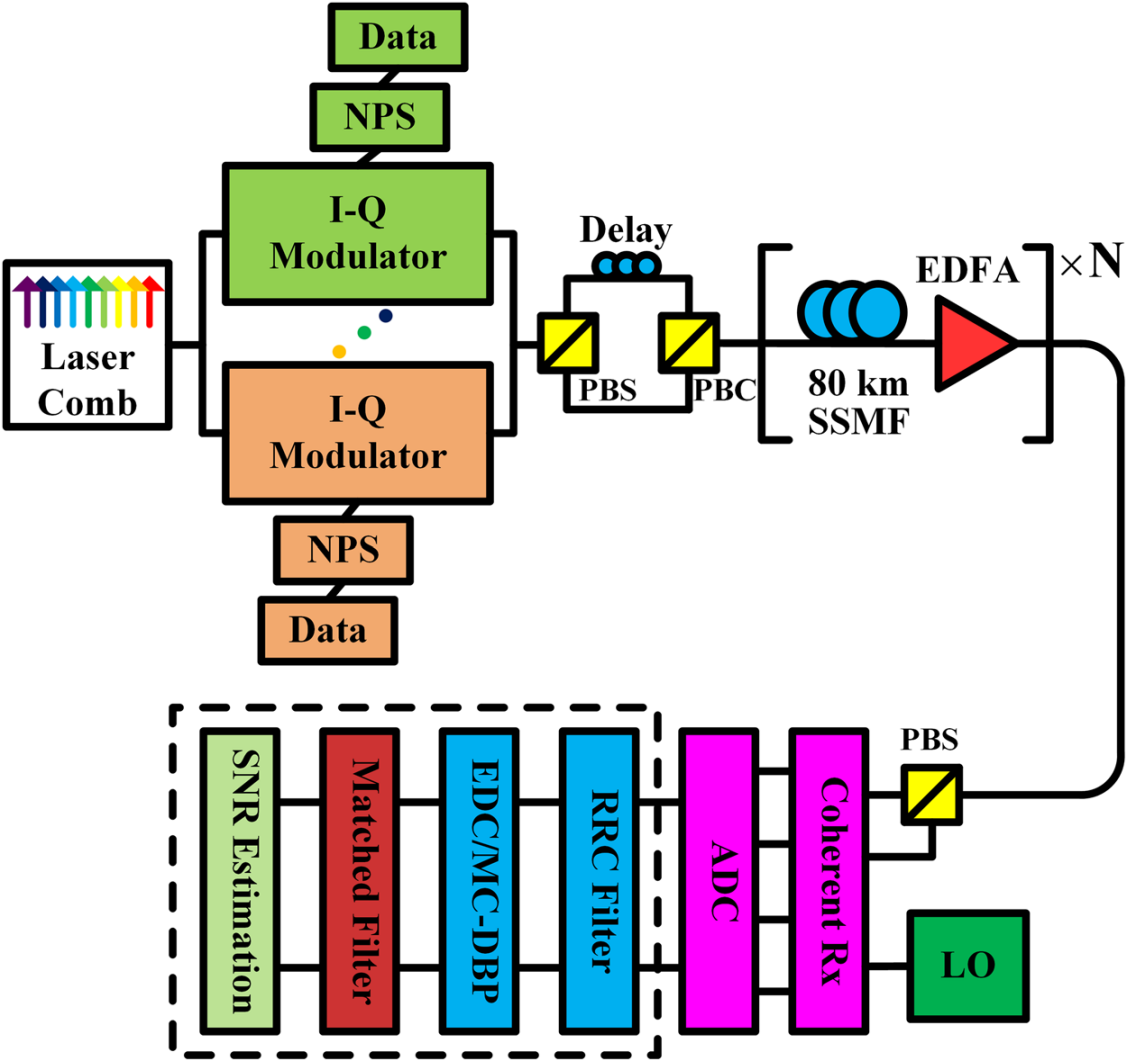
Schematic of Nyquist-spaced optical transmission system using EDC and MC-DBP. (NPS: Nyquist pulse shaping, PBS: polarisation beam splitter, PBC: polarisation beam combiner, ADC: analogue-to-digital convertor, RRC: root-raised cosine, EDC: electronic dispersion compensation).

The simulation setup of 32-Gbaud Nyquist-spaced WDM multi-channel optical transmission system is shown in Fig. 2. In the transmitter, the symbol sequence in each WDM channel is independent and random, and the symbol sequences in each polarisation are further de-correlated with a delay of half the sequence length. The Nyquist pulse shaping is implemented using a root-raised cosine (RRC) filter with a roll-off of 0.1%. The transmission link of SSMF is simulated employing a split-step Fourier solution of the Manakov equation with a logarithmic step size, and the number of steps per fibre span varies with the optical power to guarantee the accuracy^29,35^. The erbium-doped optical fibre amplifier (EDFA) in each span is included to compensate for the fibre loss. Mixed with a free-running local oscillator (LO) laser in the receiver, the signal is coherently detected with full information of in-phase and quadrature components, and the transmission impairments are compensated using the DSP modules. Detailed parameters of the optical transmission system are shown in Table 1. The laser phase noise, frequency offset and differential group delay are all neglected in the simulation. An RRC filter with a roll-off of 0.1% is applied prior to the MC-DBP module to select the desired back-propagated bandwidth, and also to reject the unwanted out-of-band ASE noise.

**Table 1:** Transmission System Parameters.

Parameter	Value
Symbol rate	32 Gbaud
Channel spacing	32 GHz
Central wavelength	1550 nm
Roll-off	0.1%
Attenuation coefficient (*α*)	0.2 dB/km
CD coefficient (*D*)	17 ps/nm/km
Nonlinear coefficient (*γ*)	1.2/W/km
Span length	80 km
EDFA noise figure	4.5 dB

## Results

In this section, the results on the performance of optical transmission and MC-DBP are described. They were obtained by evaluating the SNR of the channel of interest in SSMF links of 800 km (10 × 80 km) and 2000 km (25 × 80 km) to explore the impact of spectral broadening at different transmission distances.

### Effect of spectral broadening in optical communication systems

**Figure 3:**
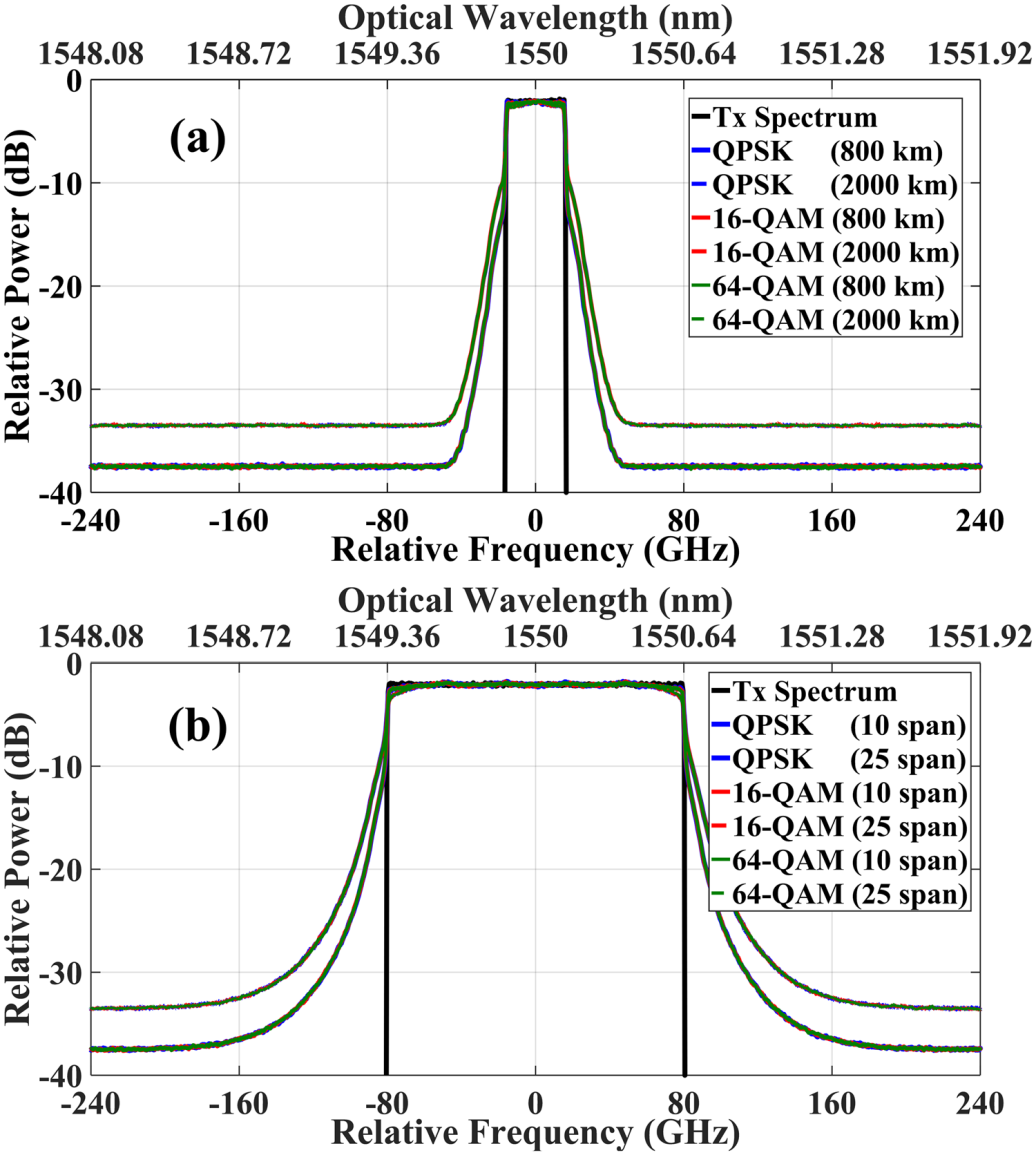
Spectral broadening effect in Nyquist-spaced optical communication systems for different modulation formats. (**a**) Single-channel system, (**b**) 5-channel system. The launched optical power is 12 dBm per channel.

The received signal spectra of the single-channel and 5-channel Nyquist-spaced optical communication systems at transmission distances of 800 km and 2000 km, are shown in Fig. 3. The optical launch power is 12 dBm per channel (slightly higher than the optimum powers of FF-DBP for both single-channel and 5-channel systems), and the modulation formats of DP-QPSK, DP-16QAM and DP-64QAM were considered. It was found that signal spectral broadening is considerable in both single-channel and 5-channel optical transmission systems at the signal power of 12 dBm per channel, both for the transmission distances of 800 km and 2000 km. Importantly, the results show that the spectral broadening effect is independent of the modulation formats.

### Impact of spectral broadening on MC-DBP

Since spectral broadening appears to be independent of modulation format, DP-16QAM system was used in all the following simulations to study the effect of spectral broadening in MC-DBP. The significance of this effect in MC-DBP was investigated in both single-channel and 5-channel optical communication systems. In traditional analyses neglecting the spectral broadening, the optimum back-propagated bandwidth in nonlinearity compensation would be set to 32 GHz for single-channel system and 160 GHz for 5-channel Nyquist-spaced transmission system.

**Figure 4:**
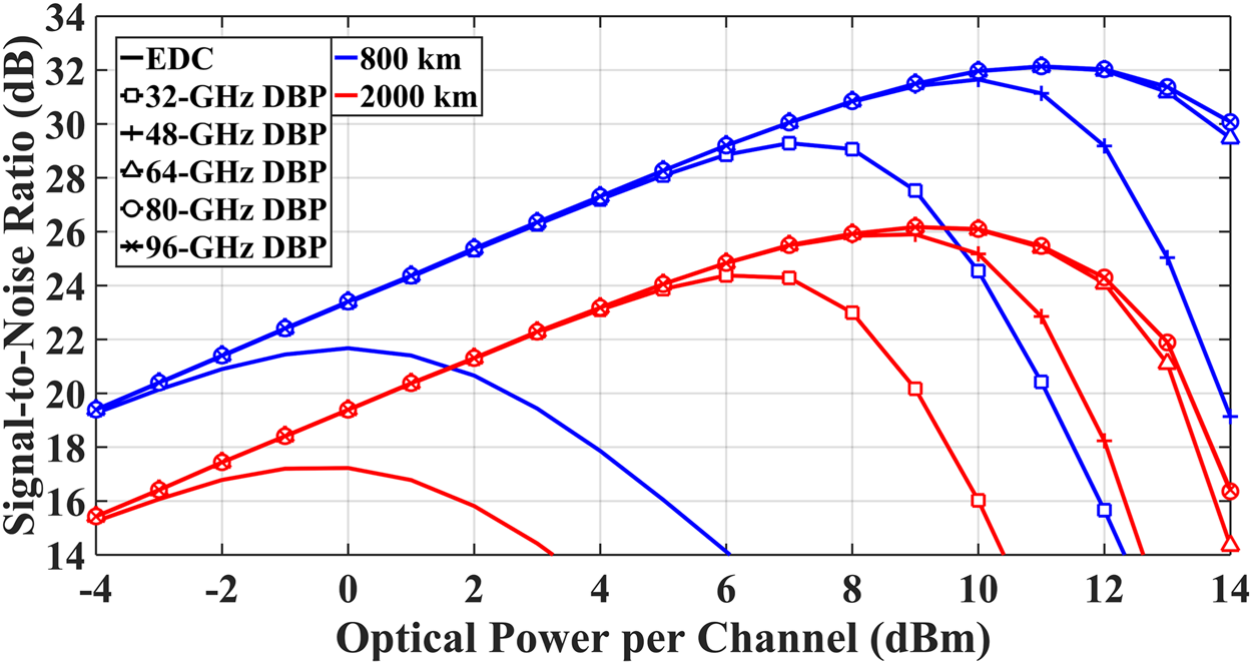
SNR versus optical signal power at different back-propagated bandwidths in MC-DBP for single-channel DP-16QAM optical transmission system.

The system performance was quantified by calculating the SNR versus optical power per channel at different back-propagated bandwidths for a single-channel (32-GHz) optical communication system. The back-propagated bandwidth was varied from 32 GHz to 96 GHz, with a step of 16 GHz. The results are shown in Fig. 4. It can be seen that the minimum back-propagated bandwidth to achieve the best SNR is 64 GHz, and the use of the transmitted signal bandwidth (32 GHz) in DBP leads to a SNR penalty of 2.8 dB (29.3 dB at 7 dBm versus 32.1 dB at 11 dBm) for 800 km transmission, and 1.8 dB (24.4 dB at 6 dBm versus 26.2 dB at 9 dBm) for 2000 km transmission.

**Figure 5:**
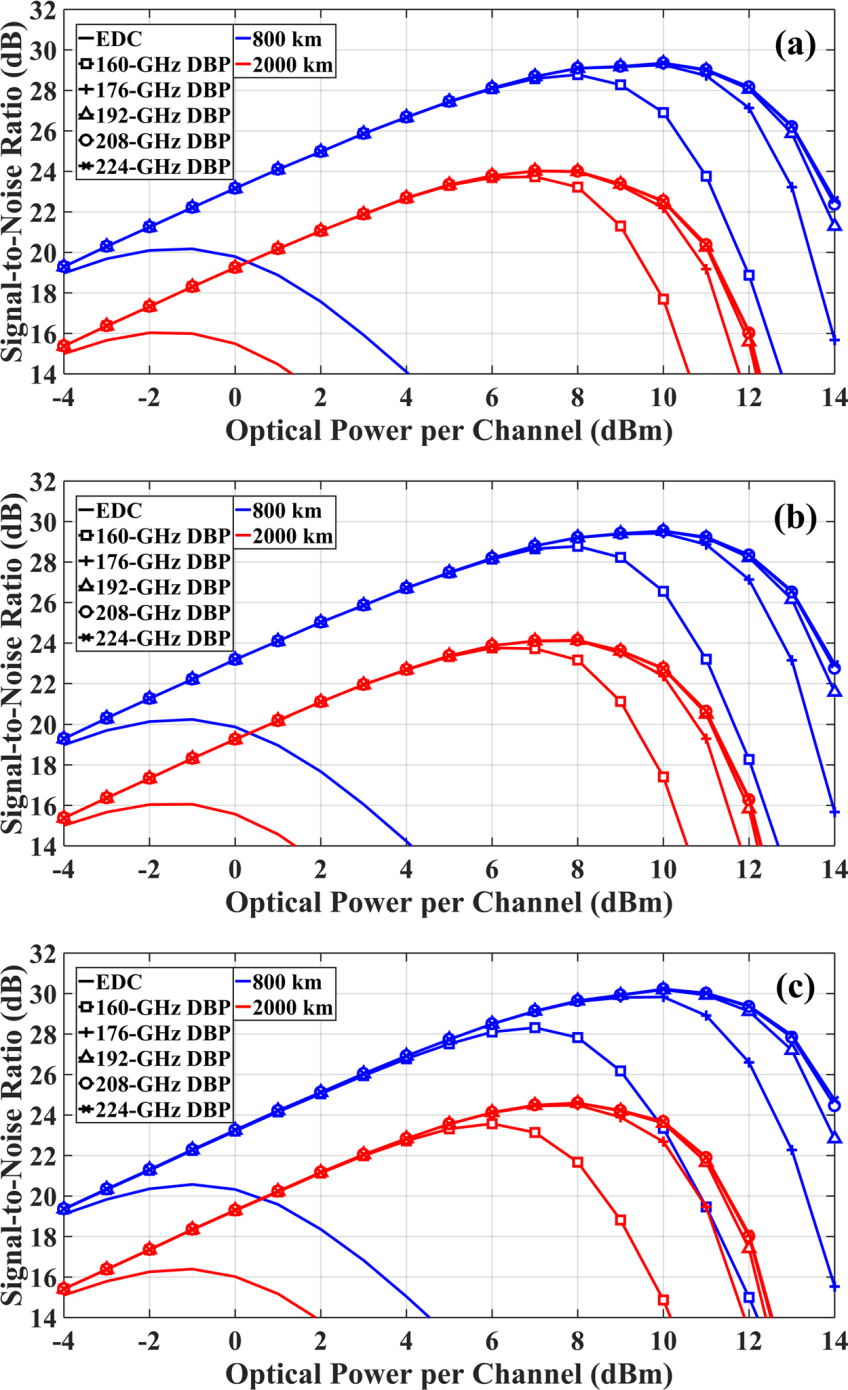
SNR versus optical signal power at different back-propagated bandwidths in MC-DBP for 5-channel DP-16QAM optical transmission system. (**a**) Central channel (channel index of 0 in Fig. 1), (**b**) second most outer channel (channel index of −1 in Fig. 1), (**c**) outer channel (channel index of −2 in Fig. 1).

Figure 5 shows the simulation results of SNR versus optical signal power per channel at different back-propagated bandwidths for MC-DBP applied to a 5-channel Nyquist-spaced optical transmission system. Since there is a symmetric distribution, the channels with indices of −2, −1, 0 (see Fig. 1) are plotted in Fig. 5 to show the properties of all channels in the 5-channel transmission system. The back-propagated bandwidth in MC-DBP varies from 160 GHz (transmitted signal bandwidth) to 224 GHz. It can be seen that the minimum back-propagated bandwidth to achieve the highest SNR is 176 GHz for the central channel and the second outer channel, and 192 GHz for the outer channel. Similar to Fig. 4, setting the compensation bandwidth equal to the transmitted signal bandwidth (160 GHz) also leads to suboptimal performance of the DBP. For the central channel, the SNR penalty is 0.6 dB (28.8 dB at 8 dBm versus 29.4 dB at 10 dBm) for 800 km transmission, and is 0.3 dB (23.7 dB at 7 dBm versus 24.0 dB at 7 dBm) for 2000 km transmission. For the outer channels, the SNR penalty is 1.9 dB (28.3 dB at 7 dBm versus 30.2 dB at 10 dBm) for 800 km transmission, and is 1.0 dB (23.6 dB at 6 dBm versus 24.6 dB at 8 dBm) for 2000 km transmission. Consistent with previous analyses, the SNR degradation (using the DBP bandwidth of transmitted signal bandwidth) due to signal spectral broadening effect is more significant for the outer channels than the central channel.

**Figure 6:**
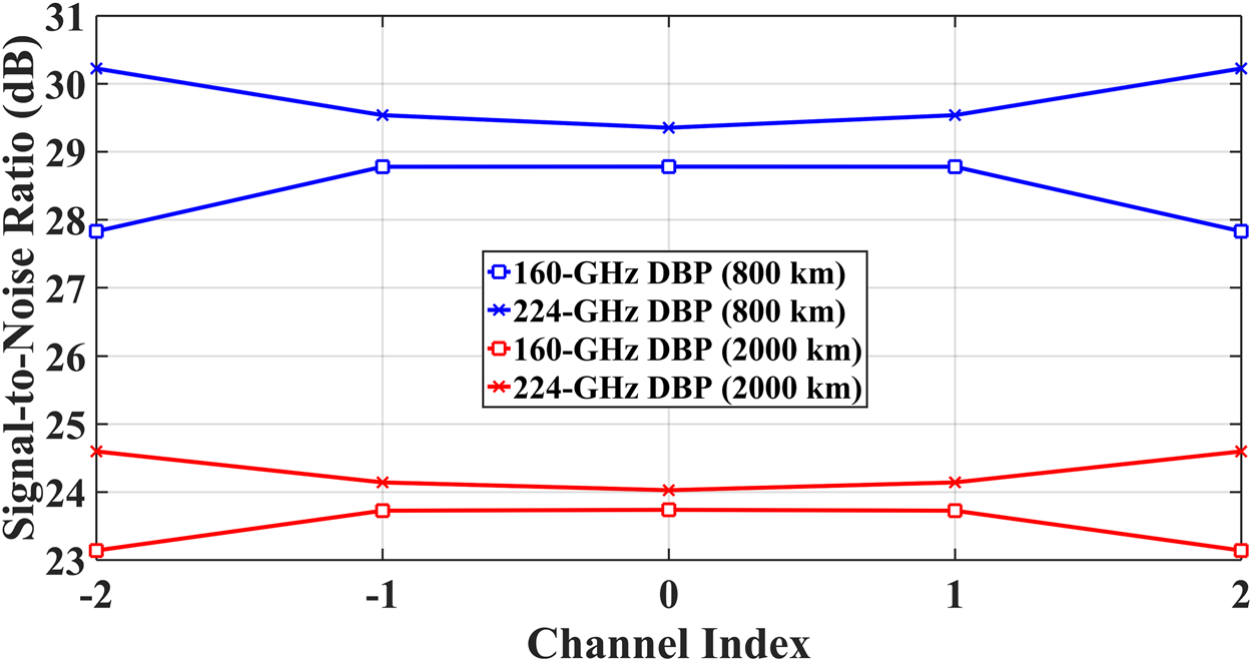
Performance degradation versus channel index in 5-channel DP-16QAM optical transmission system using MC-DBP of 160 GHz (transmitted bandwidth) and 224 GHz.

In Fig. 6, the SNR across different channels is shown for different compensation bandwidths in MC-DBP. For a compensation bandwidth of 160 GHz (the same as the transmitted signal bandwidth), the SNR penalty in MC-DBP for all channels in the 5-channel 32-Gbaud Nyquist-spaced communication system was investigated. The 224-GHz DBP was applied for realising the best DBP performance for all the channels. Unlike in Fig. 5, the optical powers used in Fig. 6 are the optimum launch power values for the central channel in both 160-GHz and 224-GHz DBP cases, corresponding to a more practical application of optical super-channel transmission. The optimum optical power is 8 dBm for 160-GHz DBP and 10 dBm for 224-GHz DBP in the case of 800 km transmission, and is 7 dBm for both 160-GHz DBP and 224-GHz DBP in the case of 2000 km transmission. Similar to Fig. 5, the degradation in DBP performance due to signal spectral broadening effect is higher for the outer channels, and is also more significant for shorter transmission distances. This is consistent with the effect shown schematically in Fig. 1, because the back-propagation using the transmitted signal bandwidth will lead to a truncation of the nonlinearly-broadened spectrum and the information loss due to the truncation is more serious for the outer channels (closer to the edge of signal spectrum). Figure 6 shows that the SNR penalty is 0.6 dB in central channel and 2.4 dB in outer channels for 800 km transmission, and the penalty is 0.3 dB in central channel and 1.5 dB in outer channels for 2000 km transmission.

**Figure 7:**
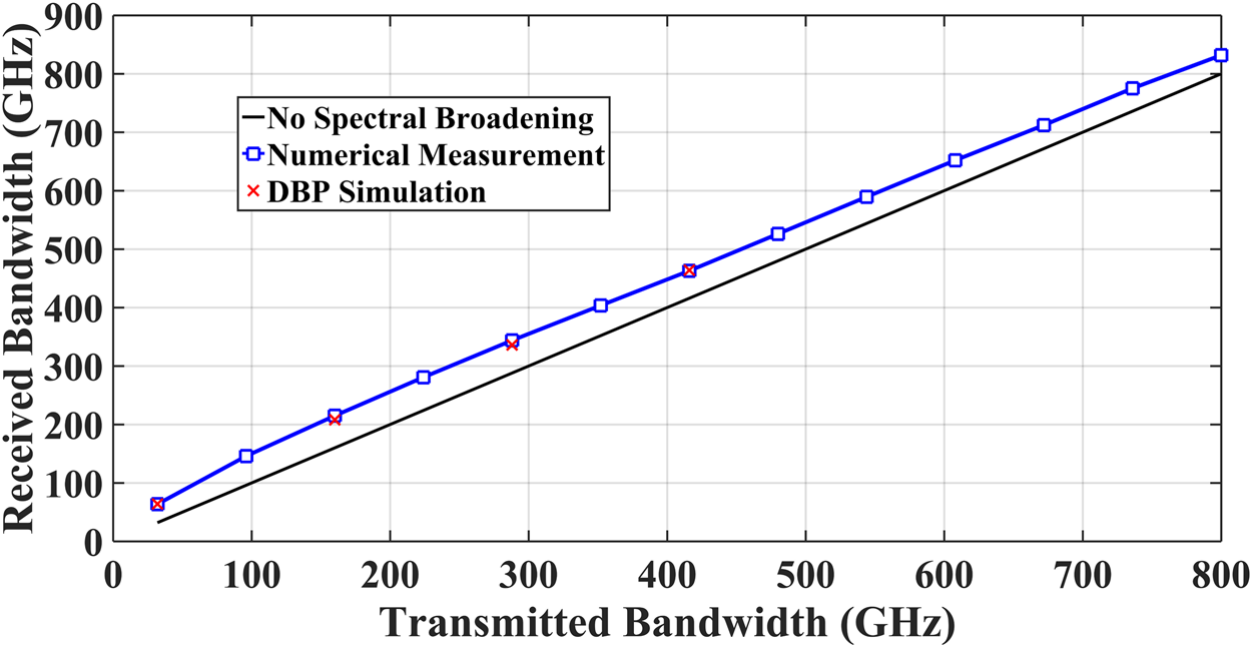
The received optical signal bandwidths considering the spectral broadening effect. Optical signal power is 12 dBm per channel, and transmission distance is 2000 km.

As shown in Fig. 7, the spectral broadening effect in Nyquist-spaced optical communication system was numerically quantified by evaluating the received signal bandwidth based on the split-step simulation of 2000 km (25 span) SSMF, varying the transmitted signal bandwidth from 32 GHz (1-channel) to 800 GHz (25-channel). Simulation parameters of the optical fibre are the same as in Table 1. The received signal bandwidth considering spectral broadening effect was assessed using two approaches. One is the above discussed the minimum compensation bandwidth for realising the optimum full-field DBP performance at a given optical launch power. The other refers to a numerical measurement of the received signal spectrum, where the received signal bandwidths (taking into account the spectral broadening) were accessed using a truncation of the broadened signal spectrum detected at the output of fibre. When 99.99% of the transmitted signal power is included, the bandwidth of the truncated spectrum is regarded as the numerically measured value of the received signal bandwidth^9,18,36^. Based on these two approaches, the received signal bandwidth considering the spectral broadening effect has been evaluated in Fig. 7, with the optical power set to 12 dBm per channel again. Firstly, it is found that the received signal bandwidth evaluated from the numerical measurement shows a good agreement with the minimum compensation bandwidth for achieving the best DBP performance at the launch power of 12 dBm per channel. The results in Fig. 7 also show that the extra bandwidth due to signal spectral broadening (in addition to the transmitted signal bandwidth) is approximately between 32 GHz and 48 GHz for all different transmitted bandwidths, at the transmission distance of 2000 km. This value gives a basic guideline for the additional guard band in both the receiver and the DSP for achieving the best performance of MC-DBP and removing all deterministic nonlinear distortions in the discussed transmission scheme.

Our work gives an insight into the effect of spectral broadening and the operation of MC-DBP for compensating all deterministic signal nonlinear interactions in the Nyquist-spaced optical communication systems. The results show that the spectral broadening effect is significant and must be taken into account to achieve the optimum performance of nonlinearity compensation, in both single-channel and multi-channel optical transmission systems. The performance of conventional MC-DBP (using the transmitted signal bandwidth as the full-field back-propagated signal bandwidth) can be further improved by considering the signal spectral broadening effect, and the improvement on the best SNR will be more significant for outer channels. Furthermore, this effect is independent of modulation formats.

## Discussion

From the above analyses, the degradation in DBP performance (for all the channels) due to signal spectral broadening effect is larger in the 800 km transmission system than that in the 2000 km transmission system. However, this does not necessarily mean that the spectral broadening effect is more significant for shorter transmission distance. It only suggests that the SNR penalty due to the spectral truncation in the nonlinearity compensation is larger in shorter distance system where the ASE noise and nonlinear distortions are relatively small. In longer-distance transmission systems, the ASE noise and nonlinear distortions are more significant than the penalty due to spectral truncation in the nonlinearity compensation, therefore the SNR degradation due to spectral broadening is less noticeable. In fact, the spectral broadening effect in optical communication systems depends on the joint impact of fibre dispersion, nonlinearities, and attenuation^37,38^.

**Figure 8:**
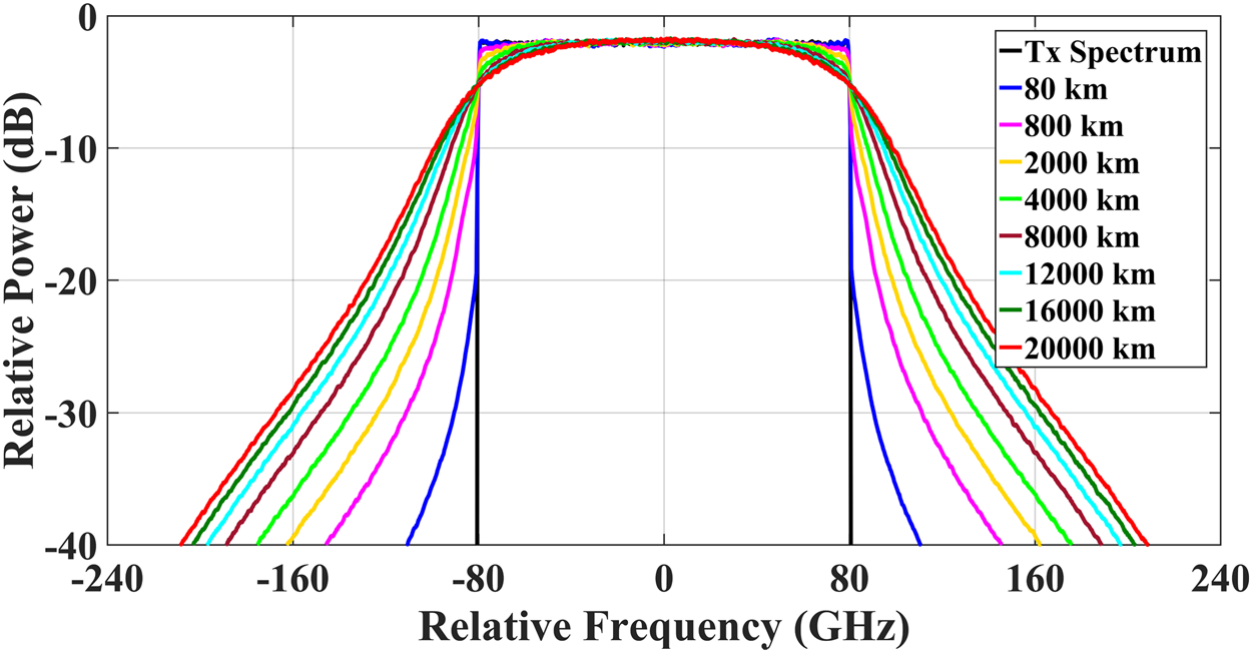
Spectral broadening effect on the received optical spectrum at different transmission distance. Optical signal power is 12 dBm per channel, and the ASE noise in optical amplifiers was not included.

The study of impact of transmission distance on the signal spectral broadening has also been carried out. To make a fair comparison between different numbers of spans, the ASE noise in the EDFA within each fibre span was not included. Figure 8 shows the received optical spectra for the 5-channel Nyquist-spaced optical transmission system with transmission distance varied from 80 km (1 span) to 20000 km (250 span). The optical launch power is again 12 dBm per channel. It is shown that the spectral broadening effect increases with the transmission distance. Interestingly, the rate of increase becomes lower for longer transmission distances, which is due to the accumulated dispersion in the transmission link gradually weakening the accumulation of the spectral broadening effect over multiple transmission spans.

It is worth mentioning that the phase noise contributions of both transmitter and LO lasers were neglected in the above studies. In practical optical transmission systems, the laser phase noise will interact with electronic dispersion compensation modules in both linear and nonlinear compensation schemes, and this will lead to an effect of equalisation enhanced phase noise (EEPN)^39–41^. The performance of high-capacity optical communication system will be further degraded by the EEPN, and the distortions will be more serious for outer channels than the central channel^42^. Specifically, considering EEPN, the performance of outer channels will be more significantly degraded on top of the penalties from the spectral broadening effect, presented in Fig. 6, compared to the central channel, in the scheme of nonlinearity compensation. Note that EEPN can be mitigated effectively using digital coherence enhancement based approaches^43,44^, while an independent measurement (or phase de-correlation) to estimate the LO laser phase fluctuation is required.

## Methods

### Theoretical analysis of spectral broadening

The spectral broadening effect due to the joint impact of dispersion, nonlinearity, and attenuation in the fibre can be described using the nonlinear Schrödinger equation written in frequency domain^10,33^

1 $$\begin{array}{c}i\frac{\partial Q(z,\omega )}{\partial z}+i\frac{\alpha }{2}Q(z,\omega )\,\,+\,\,\frac{{\beta }_{2}}{2}{\omega }^{2}Q(z,\omega )\\ \quad +\frac{\gamma }{{(2\pi )}^{3}}\iiint Q(z,{\omega }_{1})Q(z,{\omega }_{2}){Q}^{\ast }(z,{\omega }_{3})\delta (\omega +{\omega }_{3}-{\omega }_{1}-{\omega }_{2})d{\omega }_{1}d{\omega }_{2}d{\omega }_{3}=N(z,\omega ),\end{array}$$

where *Q*(*z*, *ω*) is the Fourier amplitude of the slowly-varying envelope of electric field signal in time domain $q(z,t)=\frac{1}{2\pi }{\int }_{-\infty }^{\infty }Q(z,\omega ){e}^{-i\omega t}d\omega$, where *z* is the propagation distance along the fibre, *ω* is the optical angular frequency, *α* is the fibre attenuation parameter, *β*_2_ < 0 is the fibre group velocity dispersion parameter, *γ* is the fibre nonlinear parameter, and *N*(*z*, *ω*) is the additive noise in the transmission system. However, Equation (1) has no closed-form solutions and can be solved using numerical methods such as split-step Fourier or finite-Fourier-transform methods^10^.

For the case of single-channel and zero-dispersion system, the nonlinearity-induced spectral broadening in the fibre can be analytically derived from the effect of SPM, where the evolution of the field amplitude is governed by^10,11^

2 $$q(z,t)=q(0,t){e}^{-\alpha z/2}\cdot {e}^{i{\varphi }_{NL}(z,t)},$$

where *q*(0, *t*) is the initial field amplitude and the nonlinear phase shift *ϕ*_*NL*_ is given by

3 $${\varphi }_{NL}(z,t)=\gamma {L}_{eff}{|q(0,t)|}^{2},$$

where ${L}_{eff}=(1-{e}^{-\alpha L})/\alpha$ is the effective length of the optical fibre. Hence, the angular frequency increment due to spectral broadening can be estimated as follows

4 $${\rm{\Delta }}\omega =2\gamma {L}_{eff}\langle \frac{\partial }{\partial \,t}{|q(0,t)|}^{2}\rangle ,$$

where the angle brackets stand for the time averaging. Equation (4) shows that the generation of new frequency components in the spectrum depends on the pulse shape of the input signal.

The absolute square of the Fourier transform of Eq. (2) leads to the actual spectrum *S*(*z*, *ω*) of the optical signal

5 $$S(z,\omega )\propto {|{\int }_{-\infty }^{\infty }q(0,t){e}^{i(\omega -{\omega }_{0})t+i{\varphi }_{NL}(z,t)}dt|}^{2},$$

where *ω*_0_ denotes the central angular frequency of optical signal.

### Digital signal processing in numerical simulation

In the DSP - based receiver, the EDC is carried out using frequency domain equalisation^45^, and the MC-DBP is implemented using the reverse solution of Manakov equation with a 0.5 split ratio of dispersion compensation and a logarithmic step size within each compensation span^24,35^. The number of steps per span and the nonlinear coefficient in the MC-DBP algorithm is the same as in the forward fibre propagation. An RRC filter with a roll-off of 0.1% is applied prior to MC-DBP to select the back-propagated bandwidth, and also to remove the out-of-band ASE noise. The matched filter after MC-DBP is used to select the channel of interest. The performance of each channel is assessed by estimating the SNR value over 2^18^ symbols. The oversampling rate is always set to 8 samples/symbol/channel in both the fibre propagation and DBP, to guarantee an accurate simulation of the spectral broadening effect in the system.
